# Non-Operative Management of a Common Bile Duct Injury Sustained During Cholecystectomy in a Morbidly Obese Patient. (Non-Operative Repair of CBD Injury)

**DOI:** 10.1155/1994/14936

**Published:** 1994

**Authors:** Boyd C. Ashdown, Paul V. Suhocki, Paul S. Jowell, William C. Meyers

**Affiliations:** 1 Departments of Radiology Duke University Medical Center Durham North Carolina 27710 USA; 2 Departments of Medicine Duke University Medical Center Durham North Carolina 27710 USA; 3 Departments of Surgery Duke University Medical Center Durham North Carolina 27710 USA

## Abstract

A 29 year old morbidly obese patient suffered injury to his common bile duct during cholecystectomy. Subsequent access to the biliary tree was obtained by using a long heavy gauge needle after first opacifying the system with contrast injection through a nasobiliary tube. It is now twenty six months after initial percutaneous biliary drainage placement and eighteen months after removal of all biliary access. The patient is asymptomatic and has normal liver function tests. This technique can be useful in morbidly obese patients who are at increased risk from surgical repair of biliary duct injuries.


*HPB Surgery*, 1994, Vol. 8, pp. 101–105
Reprints available directly from the publisher
Photocopying permitted by license only

© 1994 Harwood Academic Publishers GmbH
Printed in Malaysia

### Non-Operative Management of a Common Bile Duct Injury Sustained During Cholecystectomy in a Morbidly Obese Patient. (Non-Operative Repair of CBD Injury)

BOYD C. ASHDOWN*, PAUL V. SUHOCKI*, PAUL S. JOWELL† and WILLIAM C. MEYERS‡

Departments of Radiology*, Medicine† and Surgery‡, Duke University Medical Center, Durham, North Carolina 27710

A 29 year old morbidly obese patient suffered injury to his common bile duct during cholecystectomy. Subsequent access to the biliary tree was obtained by using a long heavy gauge needle after first opacifying the system with contrast injection through a nasobiliary tube. It is now twenty six months after initial percutaneous biliary drainage placement and eighteen months after removal of all biliary access. The patient is asymptomatic and has normal liver function tests. This technique can be useful in morbidly obese patients who are at increased risk from surgical repair of biliary duct injuries.

KEY WORDS: Cholecystectomy laparoscopic cholecystectomy common bile duct injury morbid obesity nasobiliary tube percutaneous biliary drainage

### INTRODUCTION

Injury to the common bile duct during cholecystectomy occurs in 0.1–0.2% of patients[1,2,3]. Due to the increased surgical risk in the obese patient[4,5], a non-operative repair was performed.

The standard technique for percutaneous biliary drainage was unacceptable due to the poor visualization of the 22 gauge Chiba needle and the inability to visualize the nondilated intrahepatic biliary radicals with injection of contrast as the needle was retracted. This modified technique involved placement of a nasobiliary tube through which the biliary system was opacified. Percutaneous access of the well visualized intrahepatic ducts followed, with an 18 gauge translumbar aortography needle. The needle provided the necessary length, rigidity and radiopacity for successfully accessing the biliary tree.

This case report emphasizes the importance of a combined effort between the endoscopist and interventional radiologist in the non-operative treatment of a patient who was at significant risk of complications related to surgery and general anesthesia.

*Address correspondence to:* Paul V. Suhocki, M.D., Department of Radiology, Box 3808, Duke University Medical Center, Durham, N.C. 27710, 919-681-2711.

### CASE REPORT

The patient is a 197 kg., 29 year old caucasian male (Figure 1) who underwent laparoscopic cholecystectomy at an outside institution for symptomatic cholelithiasis. Due to technical difficulty, the procedure was converted to a standard open cholecystectomy. Division of the common bile duct was detected prior to surgical closure. An end to end choledochostomy was performed and a #6 ureteral stent was left in the common bile duct, extending to the skin surface. On the second post-operative day, biliary drainage from the common bile duct stent had ceased. A large amount of bile began to drain from the suture line as well as two Jackson–Pratt drains (650 cc per day). On the seventh post-operative day, contrast was injected into the stent, which was seen to be no longer within the duct. It was therefore removed.

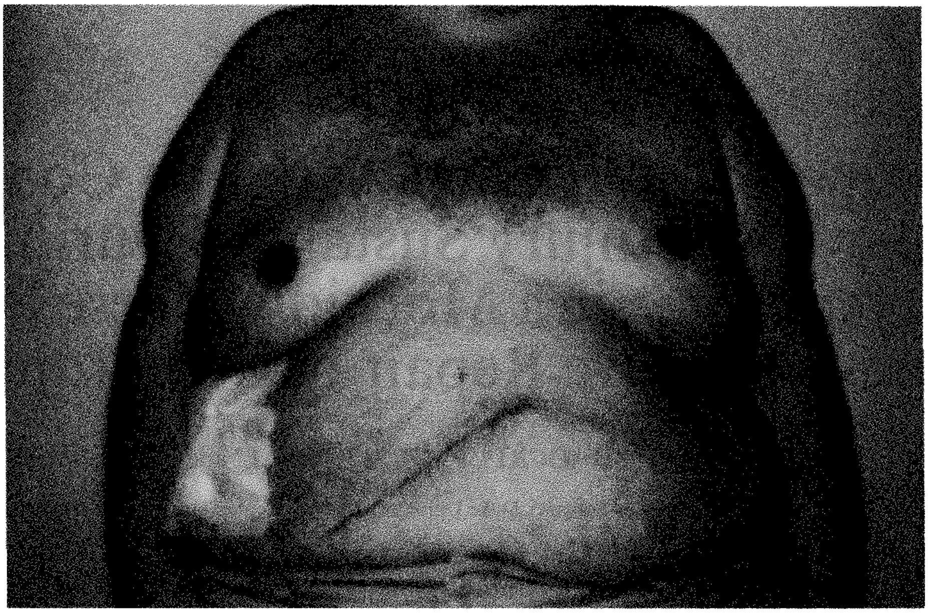

**Figure 1** Frontal view of morbidly obese male who sustained injury to the common bile duct during open cholecystectomy following conversion from laparoscopic cholecystectomy.

The patient was transferred to our institution on the ninth postoperative day. An initial ERCP demonstrated a site of contrast extravasation in the mid common bile duct (Figure 2). Attempts at retrograde passage of a guidewire across the laceration were unsuccessful. A 22 gauge Chiba needle could not be visualized on the fluoroscope when placed over the patient's right upper quadrant. The 20 cm. long needle was also felt to be too short and flexible to traverse the large amount of tissue between the skin surface and bile ducts. It was also suspected that contrast filling of non-dilated ducts would be poorly visualized during needle retraction.

A nasobiliary tube was placed with its distal portion passing through the common bile duct laceration and into the periductal space.

Contrast was then injected through the nasobiliary tube, opacifying the biliary tree as well as the periductal space at the site of bile duct injury. An 18 gauge translumbar aortography needle (Argon Medical, Athens, Texas) was inserted into the opacified right hepatic duct. A 0.035 inch angle tipped glidewire (Medi-tech Boston Scientific Corporation, Watertown, MA.) was advanced through the needle to the site of the common bile duct injury where it entered the periductal space. The needle was removed and a 5F teflon straight catheter (Universal Medical Instrument Corporation, Ballston Spa, New York) was passed. The catheter tip was lodged in the periductal space (Figure 3).

48 hours later a 0.035 in. Amplatz Super Stiff guidewire (Medi-tech Boston Scientific Corporation) was passed through the 5F catheter. A 0.018 inch Ultra-Select Nitinol guidewire, 80 cm. long with an 8 cm. angled flexible tip (Microvena Corporation, Vadnais Heights, MN) was directed across the injury site, into the distal common bile duct and into the duodenum. An 8F Ring Biliary drainage catheter (Cook) was passed, with its distal arm positioned in the duodenum (Figure 4). The catheter was placed to external drainage and output from the Jackson–Pratt drains ceased. The patient was discharged for home on the fifth post-procedure day with the biliary drainage tube to external drainage. Contrast extravasation from the site of injury was seen until 16 weeks following the drainage procedure, at which time the catheter was converted to internal drainage. Because of a stenosis seen at the site of injury one month later, the common bile duct was dilated with a 10 mm diameter, 4 cm long balloon (Meadox Surgimed Inc., Oakland, NJ). A 14F Cope biliary drainage catheter (Cook) was placed and left to internal drainage. Two months later, the drainage catheter was replaced with a 5F Multipurpose catheter (Cook), the tip of which was positioned peripherally in an intrahepatic duct. This was used as an emergency access catheter while the patient was given a trial of

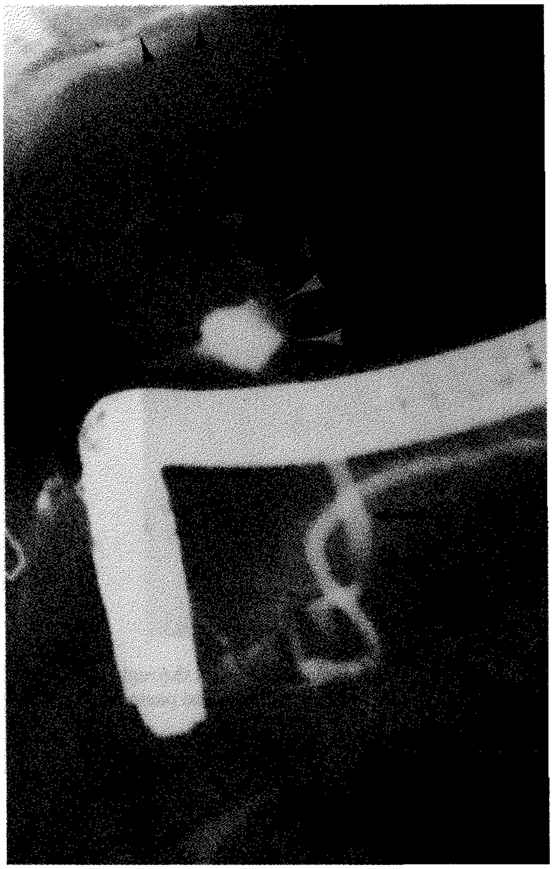

**Figure 2** FRCP demonstrated extravasation of contrast (white arrowheads) from the common bile duct (black arrows). Contrast has traveled retrograde into the right intrahepatic ducts (black arrowheads).

internal drainage. The patient continued to do well and the access catheter was removed 6 weeks later. The patient remains asymptomatic, with normal liver function tests, at 18 months following catheter removal.

### DISCUSSION

Injury to the common bile duct is the most common complication of cholecystectomy and can be difficult to correct. Common bile duct injury occurs most frequently during open cholecystectomy when it is mistaken for the cystic duct, resulting in resection of part of the common bile duct and/or the common hepatic duct[6]. The injury in this morbidly obese man occurred during open cholecystectomy and consisted of simple division of the common bile duct. The surgical procedure was difficult because of the patient's size, as was subsequent radiologic management. Non-operative treatment of the common bile duct injury was chosen after transfer of this patient because it was felt safer than an open repair. For obese patients, the mortality associated with surgery has been reported as being as high as 6%; this is felt to be caused by: 1) altered respiratory and cardiovascular physiology making maintenance of and recovery from general anesthesia difficult, 2) protracted time of operation and 3) increased post-operative incidence of wound infection, deep venous thrombosis and pulmonary embolus[4,5]. This patient also seemed to have an intact biliary system which was simply leaking and/or stenosed.

Opacification of the non-dilated biliary tree via nasobiliary tube provided a target, the right hepatic duct, for aiming the needle. This step also reduced the risk of complications such as hepatic artery pseudoaneurysm, associated with multiple needle passes with a large gauge needle. We were not sure that contrast would travel retrograde through the injured duct, but this step obviated the need for multiple blind needle passes.

The first description of an 18 gauge used for percutaneous transhepatic cholangiography was in 1937[7] and in 1969 for biliary drainage[8]. The "skinny needle" technique, using a 22 gauge needle, began in 1974[9]. Our aortography needle provided the length, rigidity and radiopacity necessary for this patient. The needles currently marketed for biliary drainage do not provide all these features. The increased risk of hemorrhage and bile peritonitis with an 18 gauge needle (as high as 25%[10]) compared with the 5–7.9% rate with a 22 gauge needle[11] prohibits its routine use.

A Wallstent was not placed for the benign stenosis which developed 5 months post-procedure because of the satisfactory anatomic and physiologic response to balloon dilatation and the current lack of data on long term patency of Wallstents. Further intervention can be performed endoscopically in a retrograde fashion if stenosis recurs.

Our patient sustained a common bile duct injury following conversion from laparoscopic cholecystectomy to open cholecystectomy. Technical difficulties limited the laparoscopic approach. The laparoscopic method is now considered the procedure of choice in most patients requiring cholecystectomy[12]. It was first described in 1989 by Dubois in France[13], and in the southern United States by Reddick[14]. Intraoperative conversion of a laparoscopic to conventional open cholecystectomy is a complication that occurs with an incidence of 4.3 percent[1]. In a prospective analysis of 1,518 laparoscopic cholecystectomies, the Southern

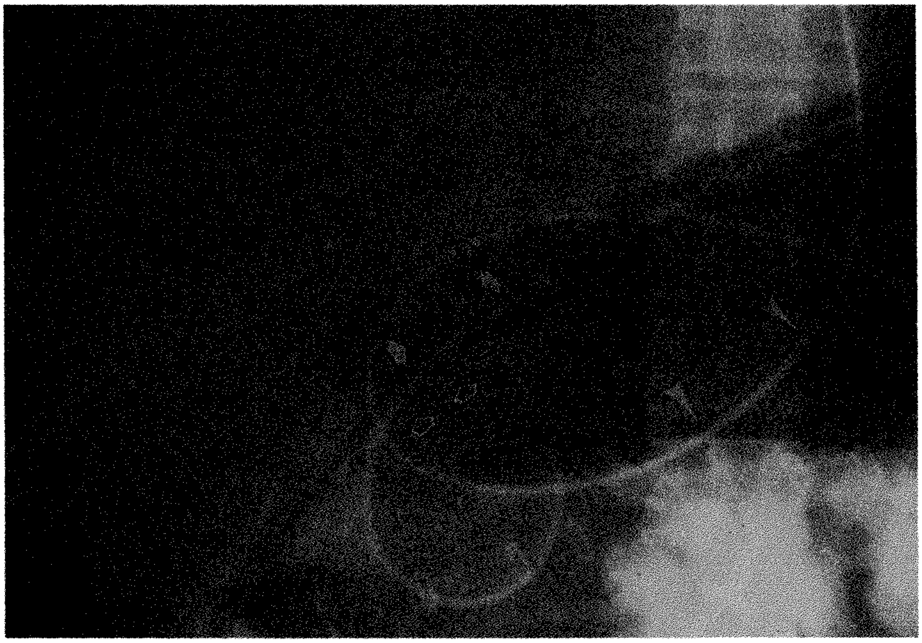

**Figure 3** A nasobiliary tube is coiled in the stomach (closed arrowheads). The distal tip (closed arrows) has passed through the site of CBD injury and is coiled in the adjacent tissues. The tip of the percutaneously passed straight catheter (open arrows) has also passed through the site of injury and lies in the periductal tissues.

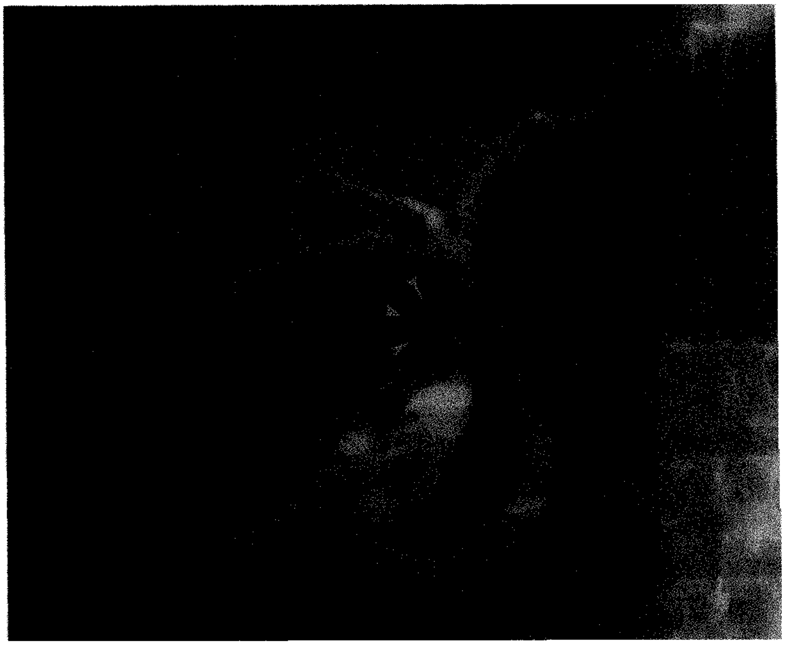

**Figure 4** The nasobiliary tube has been removed. A Ring biliary drainage catheter has been passed into the duodenum. Contrast injection of the catheter demonstrates extravasation (arrowheads) from the CBD.

Surgeons Club reported a 2.2 percent incidence of CBD injury among the first 13 operations performed by a surgeon, subsequently decreasing to 0.1 percent[1]. This is compared with a probable 0.1–0.2 percent bile duct injury during conventional cholecystectomy[2,3,15]. As more surgeons perform laparoscopic cholecystectomy, the interventional radiologist will be involved more frequently in the management of common bile duct injuries. The laparoscopic biliary injury tends to be more serious than the open one, and this technique is unlikely to be applicable for permanent results. However, the method described can be used for placement of tubes in a number of such cases in the morbidly obese patient.

### REFERENCES

1. The Southern Surgeons Club (1991) A prospective analysis of 1,518 laparoscopic cholecystectomies performed by Southern U. S. surgeons. *N. Engl. J. Med.*, **324**, 1073–1078.
2. Viikari, S. J. (1960) Operative injuries to the bile ducts. *Acta. Chir. Scand.*, **119**, 83–92.
3. Meyers, W. C., Jones, R. S. (1990) *Textbook of Liver and Biliary Surgery*. J. B. Lippincott, Philadelphia, pp. 373–390.
4. Sabiston, D. C. (1991) *Textbook of Surgery*. W. B. Saunders, Philadelphia, pp. 928–94.
5. Fisher, A., Waterhouse, T. D., Adams, A. P. (1975) Obesity: its relation to anaesthesia. *Anesthesiology*, **30**, 633–647.
6. Davidoff, A. M., Pappas, T. N., Murray, E. A., Hilleren, D. J., Johnson, R. D., Baker, M. E., Newman, G. E., Cotton, P. B., Meyers, W. C. (1992) Mechanisms of major biliary injury during laparoscopic cholecystectomy. *Ann. Surg.*, **215**, No. 3: 196–202.
7. Huard, P., Do-Xuan-Hop (1937) La Ponction Transhepatique des Canaux Biliares. *Bull. Soc. Med-Chir.*, Indochine, **15**, 1090.
8. Kaude, J. V., Weidenmier, C. H., Agee, O. F. (1969) Decompression of bile ducts with the percutaneous transhepatic technique. *Radiology*, **93**, 69–71.
9. Okuda, K., Tanikawa, K., Emura, T., Kuratomi, S., Jinnonchi S., Urabe, K., Sumikoshi, T., Kanda, Y., Fukuyama, Y., Musha, H., Mori, H., Shimokawa, Y., Yakushiji, F., Matsuura, Y. (1974) Nonsurgical, percutaneous transhepatic cholangiography – diagnostic significance in medical problems of the liver. *Dig. Dis.*, **19**, 21–36.
10. Ferrucci, J. T., Wittenberg, J., Sarno, R. A., Dreyfuss, J. R. (1976) Fine needle transhepatic cholangiography: A new approach to obstructive jaundice. *AJR*, **127**, 403–407.
11. Jain, S., Long, R. G., Scott, J., Dick, R., Sherlock, S. (1977) Percutaneous transhepatic cholangiography using the "Chiba" needle – 80 cases. *Br. J. Radiol.*, **50**, 175–180.
12. Schirmer, B. D., Edge, S. B., Dix, J., Hyser, M. J., Hanks, J. B., Jones, R. S. (1991) Laparoscopic cholecystectomy: Treatment of choice for symptomatic cholelithiasis. *Ann. Surg.*, **213**, 665–677.
13. DuBois, F., Icard, P., Berghelot, G., Levard, H. (1990) Coelioscopic cholecystectomy: Preliminary report of 36 cases. *Ann. Surg.*, **211**, 60–62.
14. Reddick, E. J., Olsen, D. O. (1989) Laparoscopic laser cholecystectomy: A comparison with mini-lap cholecystectomy. *Surg Endosc.*, **3**, 131–133.
15. Raute, M., Schaupp, W. (1988) Iatrogenic damage of the bile ducts caused by cholecystectomy. *Langenbecks Arch Chir.*, **373**, 345–354.

